# One-year follow-up of the new European reference network for pediatric cancers (ERN PaedCan) tumor board for pediatric CNS tumors: lessons learnt and future prospects

**DOI:** 10.1007/s11060-025-05189-5

**Published:** 2025-09-16

**Authors:** Marthe Sönksen, Brigitte Bison, Lisa Bußenius, Jelena Rascon, Denise Obrecht-Sturm, Barry Pizer, Katrin Scheinemann, Martin Schalling, Ruth Ladenstein, Martin Mynarek, Stefan Rutkowski

**Affiliations:** 1 Pediatric Hematology and Oncology, University Medical Center Hamburg-Eppendorf, Martinistr. 52, 20246 Hamburg, Germany; 2 Diagnostic and Interventional Neuroradiology, Faculty of Medicine, University of Augsburg, Augsburg, Germany; 3 Center for Pediatric Oncology and Hematology, Vilnius University Hospital Santaros Klinikos, Vilnius, Lithuania; 4 Faculty of Medicine, Vilnius University, Vilnius, Lithuania; 5 Department of Pediatric Oncology, Alder Hey Children’s NHS Foundation Trust, Liverpool, UK; 6 Pediatric Hematology-Oncology Center, Children’s Hospital of Eastern Switzerland, St Gallen, Switzerland; 7 Faculty of Health Science and Medicine, University Lucerne, Lucerne, Switzerland; 8 St Anna Children’s Cancer Research Institute CCRI, Vienna, Austria

**Keywords:** Childhood cancer, European Reference Networks (ERN), European Reference Network for pediatric cancers (ERN PaedCan), Pediatric brain tumors, Multidisciplinary tumor boards

## Abstract

**Purpose:**

European Reference Networks (ERN) are collaborative networks connecting healthcare professionals across Europe. A virtual multidisciplinary tumor board (VMTB) for children with central nervous system (CNS) tumors was established within the ERN for pediatric oncology (ERN PaedCan) in 2022. We report the experience with this new format.

**Methods:**

A web-based questionnaire was distributed to physicians for cases presented between November 2022 and November 2023, addressing the implementation of provided recommendations, satisfaction and basic data about the local institution. Baseline information of the presented cases was taken from anonymized VMTB protocols.

**Results:**

In the first year, 19 patients from 11 institutions located in nine European countries were discussed in 21 VMTB. The German national reference center for neuroradiology demonstrated MRI findings in 19/21 (91%) conferences. 19 questionnaires were answered by physicians from all participating countries. Main reason for VMTB presentation were questions about therapy (79%). Presenting institutions treated a median of 10 (5–150) neuro-oncological pediatric patients per year. All hospitals conducted own institutional tumor boards. National central review was available in 3/9 countries (33%). Recommendations were followed, at least partly, in all except one patient experiencing unexpected clinical deterioration. Recommendations were considered helpful in 90%. All participants would recommend the VMTB to colleagues. Technical issues regarding data provision were reported as the main obstacle in 56%.

**Conclusion:**

A European VMTB for pediatric patients with CNS tumors is feasible and perceived as useful by the participants. Recommendations were followed frequently. Optimization of privacy-compliant data exchange is crucial for continuance of the format.

**Supplementary Information:**

The online version contains supplementary material available at 10.1007/s11060-025-05189-5.

## Introduction

Central nervous system (CNS) neoplasms are the second most frequent cancer type in children [1, 2]. In Europe, it is estimated that almost 1,900 children and young adults below the age of 20 were diagnosed with CNS tumors in 2022, corresponding to an estimated incidence of 2/100,000 [3].

The number of registered cases is unequally distributed across European countries, ranging from over 1,700 cases in 5 years (France) to as little as 8 cases in 5 years (Cyprus) [1]. Despite improvement of overall survival of European pediatric CNS tumor patients over the last decades, the geographical disparities regarding survival of children with CNS tumors among European countries persist [1].

Treatment of pediatric cancer patients depends on the medical infrastructure and expertise of the respective country. Due to low case numbers, pediatric oncologists in some centers may treat certain pediatric CNS tumor entities only few times, if any, each year. Therefore, external expert advice may help them take optimal decisions for diagnostic assessments, options for treatment and open clinical trials which might be available for these indications. Former reports indicate that international expert consultation may indeed provide beneficial assistance in the care of children with cancer [4–7].

European Reference Networks (ERNs) are virtual networks between healthcare professionals across Europe [8]. ERNs bring together experts on rare and complex diseases with the aim to make their expertise accessible to other European healthcare providers. Following the Directive 2011/24/EU of the European Parliament and of the Council on the application of patients’ rights in cross-border healthcare (CBHC) [9], the ERN for pediatric cancer (ERN PaedCan) was established in 2017 [8, 10]. Its aim is to improve survival and quality of life of children with cancer by reducing existing inequalities among member states of the European Union (EU), in close collaboration with the European Society for Pediatric Oncology (SIOPE) and the European Clinical Trial Groups. Currently, ERN PaedCan unites institutions from all 27 EU member states plus Norway (21 countries as full members, 7 as affiliated partners) [11]. The European community has identified the implementation of cross-border case discussions in virtual multidisciplinary tumor boards (VMTB) as a cornerstone of knowledge sharing, allowing information to travel to the patients and their treating physicians instead of vice versa. Virtual consultations for rare pediatric tumors have been successfully conducted within the predecessor project ExPO-r-NeT (European Expert Pediatric Cancers Reference Networks for Diagnostics and Treatment) via chat [4]. While a chat format provides certain benefits such as around-the-clock accessibility to the discussion, video conferences with direct interaction between treating physicians and consulting experts and the possibility of real-time demonstration of radiological data or histological slides may help strengthen quality and conciseness of discussions. A secure IT platform, the Clinical Patient Management System (CPMS), was developed by the EU to enable data exchange compliant with the General Data Protection Regulation (GDPR) for virtual CBHC consultations within ERNs [12]. Predefined expert groups on five pediatric cancer entities (CNS tumors, Ewing sarcoma, osteosarcoma, rare tumors, soft tissue sarcoma) have been established on CPMS within ERN PaedCan in the first pilot phase to manage incoming requests and to set up the actual VMTB for the respective entity [13]. We here report on the experiences of the SIOPE-BTG (Brain Tumor Group of the European Society for Pediatric Oncology) with the pilot phase of the new ERN PaedCan CPMS tumor board for CNS tumors.

## Methods

The ERN PaedCan CNS VMTB was established in September 2022 and the European community was informed about it via a SIOPE-BTG newsletter and at SIOPE-BTG meetings in 2022 and 2023. The first VMTB discussion took place in November 2022. VMTB were scheduled weekly on Tuesdays at 11 am Central European Time and held on demand as video conferences, with a maximum of two slots and 60 min of discussion time per week. All written communication and the VMTB discussion itself were conducted in English. Participation of the inquiring physician was mandatory. After registration of their case with the VMTB coordinator, inquiring physicians created a panel for their case on the CPMS prior to the VMTB discussion. Informed consent from patients or legal guardians for sharing pseudonymized clinical information had to be obtained by the inquiring physician preceding panel creation. Any person-identifying data had to be removed before uploading clinical information onto CPMS. Instead, inquiring physicians were able to identify their patient by panel ID. Magnetic resonance imaging (MRI) data were also uploaded pseudonymized. Data had to be uploaded two working days prior to tumor board discussion to enable sufficient preparation of the consulting experts. The VMTB coordinator reviewed completion and appropriateness of clinical data prior to the video conference to enable meaningful and efficient consultations. The inquiring physician presented the case during the video conference without any person-identifying data. Members of the SIOPE-BTG with particular expertise for the respective tumor entity were invited as experts by the VMTB coordinator and offered their expertise voluntarily and free of charge. Attendance of the video conference was possible for members of the inquiring institution, consulting experts and the coordinators. After the discussion, written minutes summarizing the case information, the discussion during the VMTB conference, and recommendations were provided by the board leadership team.

All physicians presenting patients in the ERN PaedCan CNS tumor board between November 1, 2022 and November 30, 2023 were asked to participate in the survey. An online questionnaire using LimeSurvey version 6.5 (LimeSurvey GmbH, Hamburg, Germany) was created and distributed to physicians via e-mail in December 2023. Questions addressed the reason for presentation in the ERN PaedCan CNS tumor board, the implementation of recommendations, the satisfaction with the ERN PaedCan CNS tumor board and general information about the inquiring institution. A reminder was sent four weeks after the initial request to increase response rate. Participation was voluntary and there was no financial compensation. Each participating physician received a key word for protected completion of the survey and to allow attribution to their institution. Descriptive analyses were conducted using R v4.3.1 (R Core Team, Vienna, Austria) and SPSS Version 29 (IBM Corp., Armonk, NY, USA). The graphic depiction of participating countries was created using mapchart.net.

## Results

**Fig. 1:**
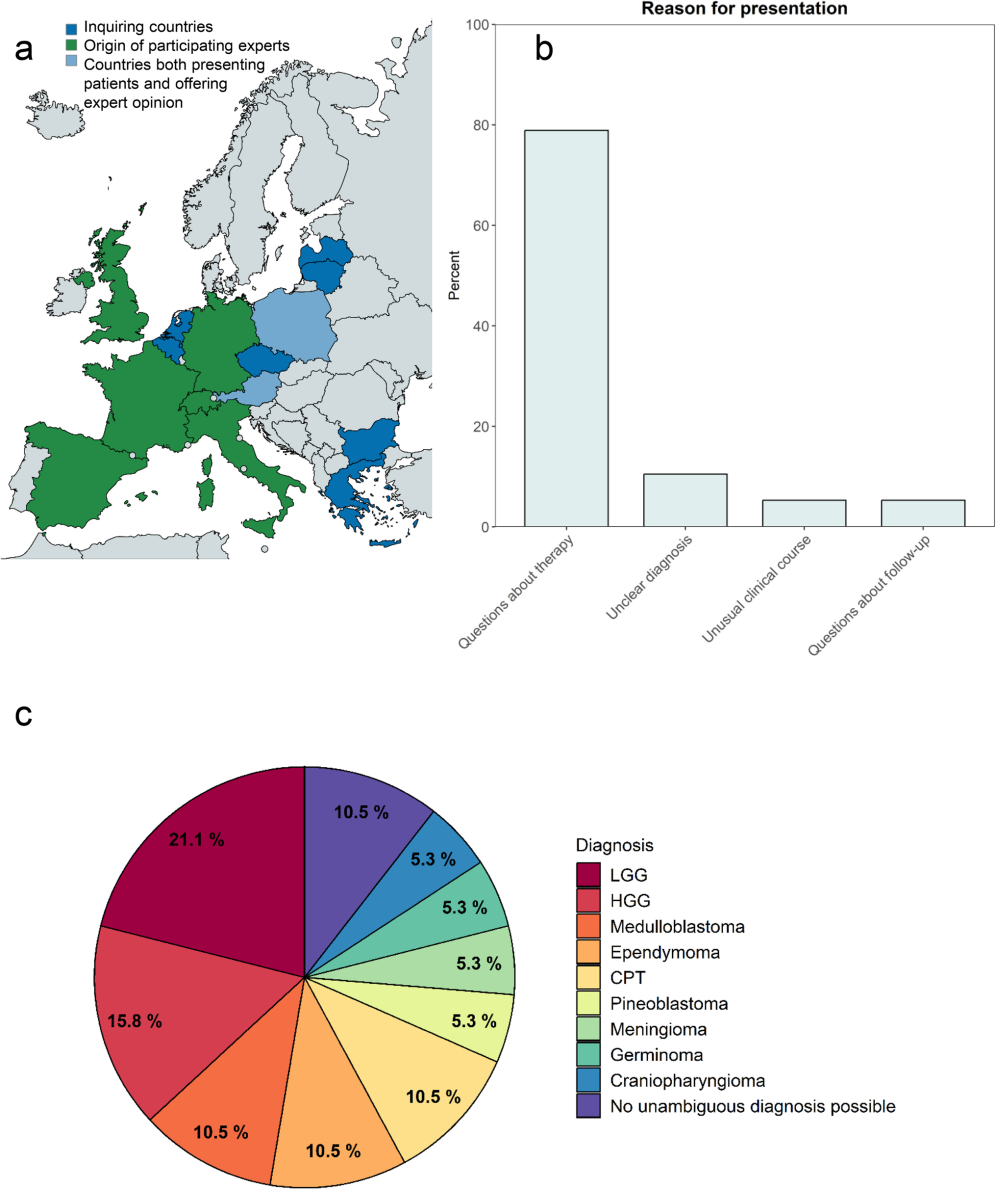
**a** European countries participating in the ERN PaedCan CNS tumor board from November 2022 – November 2023 **b** Main reason for presentation in the ERN PaedCan CNS tumor board according to the inquiring physician **c** Diagnoses of patients presented in the ERN PaedCan CNS tumor board

In the first year, 19 patients from 11 hospitals located in nine European countries were discussed in the ERN PaedCan CNS tumor board in 21 virtual tumor boards (Table 1). Consulting experts came from eight European countries (Fig. 1a). Inquiring physicians from all nine countries answered 19/21 questionnaires (90.5%). All countries were high-income countries (HIC) according to the World Banks classification 2023–2024 [14]. Almost half of the cases were presented by one institution (8/19 patients were discussed in 9/21 VMTB), while the other hospitals presented 1–2 cases each (Table 1).

**Table 1:** Characteristics of cases presented in the ERN PaedCan CNS tumor board

Characteristics	Number of patients (% of patients)
Treating country (*institution*)	
Lithuania (*Vilnius University Hospital Santaros Klinikos*)	8 (42.1)^*a*^
Austria (*Medical University of Graz*)	2 (10.5)
Czech Republic (*University Hospital Motol, Prague* / *University Children’s Hospital Brno*)^*b*^	2 (10.5)
Greece (*'Aghia Sofia' Children's Hospital, Goudi, Athens* / *Ippokratio General Hospital of Thessaloniki*)^*b*^	2 (10.5)
Belgium (*Antwerp University Hospital*)	1 (5.3)
Bulgaria (*University Hospital Varna*)	1 (5.3)^*c*^
Latvia (*Children's Clinical University Hospital Riga*)	1 (5.3)
Netherlands (*Princess Maxima Center Utrecht*)	1 (5.3)
Poland (*Children’s Memorial Health Institute, Warsaw*)	1 (5.3)
Gender	
Female	7 (36.8)
Male	12 (63.2)
Median Age (Range [years])	10 (1–17)
Time point of presentation	
At initial diagnosis	9 (47.4)
- No adjuvant therapy initiated	− 5
- Adjuvant therapy initiated < 6 weeks from discussion	− 4
During therapy	1 (5.2)
At relapse/PD	9 (47.4)
Presence of tumor predisposition syndrome	
Yes, confirmed	5 (26.3) - 1 × DICER1, 1 × NF1, 2 × LFS, 1 × SCID
No, but further diagnostics recommended due to case constellation	3 (15.8)
No, case details not suggestive for TPS	11 (57.9)
Images presented by neuroradiologist from the German national reference center for neuroradiology	
Yes	19 (90.5)
No	2 (9.5)

^*a*^One of these patients was discussed twice

^*b*^Each institution presented one case, respectively

^*c*^This patient was discussed twice

NF: neurofibromatosis, LFS: Li-Fraumeni syndrome, SCID: severe combined immunodeficiency

### Characteristics of case discussions

Case characteristics are summarized in Table 1. Nine different tumor entities were discussed, reflecting the diversity of pediatric neuro-oncology. Low-grade glioma was the most frequently discussed tumor entity (n = 4), followed by high-grade glioma (n = 3), medulloblastoma, ependymoma and choroid plexus tumors (CPTs) (each n = 2; Fig. 1c). In one of these patients, the initial diagnosis was changed from embryonal tumor with multilayered rosettes to ependymoma due to molecular diagnostics conducted in another EU country upon recommendation in the VMTB. In two patients, the tumor could not be assigned to a known brain tumor entity at the time of the discussion despite extensive neuropathological diagnostics and second opinions. Around a quarter of patients had a tumor predisposition syndrome (TPS) (26.3%, n = 5) and in another 15.8%, further diagnostics regarding TPS were recommended (Table 1). Of the nine patients discussed at initial diagnosis (47.4%), two were discussed twice because of uncertain histological diagnosis at initial presentation. Five had not received any other treatment than surgery at time of discussion. Four patients were discussed within the first six weeks after initiation of adjuvant therapy. Almost half of the patients were presented at relapse or progressive disease (PD) (47.4%, n = 9). One patient experiencing unexpected side effects of treatment was discussed during therapy. The main reasons to request discussion according to inquiring physicians were treatment-related questions (n = 15/19), followed by unclear diagnosis (n = 2/19), unusual clinical course (n = 1/19) and questions regarding a follow-up strategy (n = 1/19; Fig. 1b).

### General information about inquiring institutions and countries

Inquiring institutions treated a median of 10 (min. 5; max. 150) neuro-oncological pediatric patients per year. All hospitals conducted their own institutional tumor boards (Supplementary table S1). For one institution, international tumor boards such as the ERN PaedCan CNS tumor board were the only option to receive pan-institutional advice on patients. National central review was available in three countries (33.3%): in two countries, neuropathology, MRI images and cerebrospinal fluid (CSF) cytology could be centrally reviewed, and in another country, neuropathology national central review was available (Supplementary table S1).

### Specialties involved in the ERN PaedCan CNS tumor board

Pediatric oncologists with particular expertise regarding the entities discussed and neuroradiologists participated in all ERN PaedCan CNS tumor board discussions (Table 2). Other specialties were frequently involved where needed and appropriate. A median of 3 (range 2–5) different specialties were present at tumor board discussion (Supplementary table S2). Radiotherapists participated in 12/21 discussions (57.1%), followed by neurosurgeons and neuropathologists, which participated in a third of discussions each (n = 7/21). An expert neuroradiologist from the neuroradiological reference center for the pediatric brain tumor (HIT) studies of the German Society for pediatric oncology and hematology (GPOH) demonstrated MRI findings in 19/21 conferences (90.5%). Reference assessment differed from the local institution’s report in four cases (Supplementary Table S3). In six cases, diagnostic imaging did not meet the minimum criteria defined within the recommendations by the SIOPE-BTG imaging working group [15], and repetition of imaging due to this circumstance was recommended in two cases (Supplementary Table S4). In two conferences, a local neuroradiologist demonstrated the MRI images due to inability of Digital Imaging and Communications in Medicine (DICOM) data exchange via CPMS prior to the tumor board for technical reasons.

**Table 2:** Involvement of different specialties in the ERN PaedCan CNS tumor board

Specialty	Number of tumor board discussions attended (% of discussions)
Pediatric oncology	21 (100)
Neuroradiology	21 (100)
Radiotherapy	12 (57.1)
Neurosurgery	7 (33.3)
Neuropathology	7 (33.3)
Ophthalmology	3 (14.3)

### Recommendations and implementation of recommendations

**Fig. 2:**
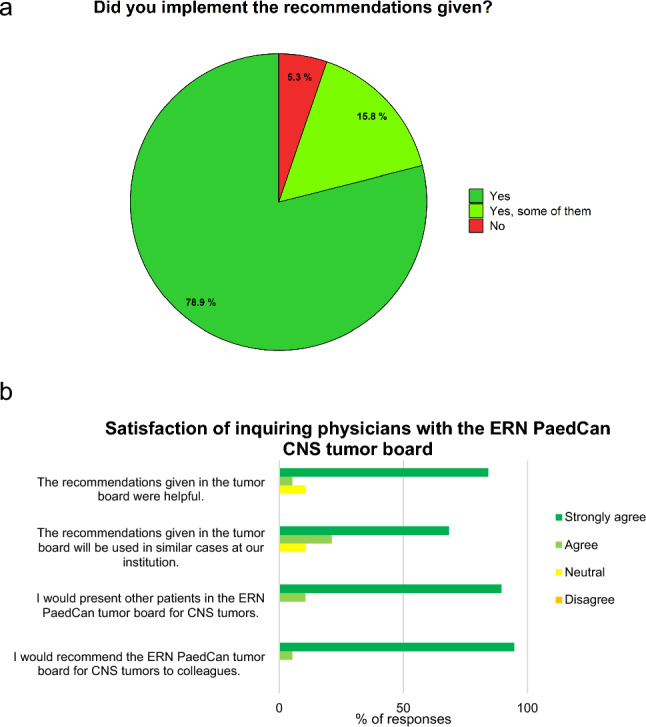
Implementation of recommendations **a** and satisfaction of inquiring physicians with the ERN PaedCan CNS tumor board **b**, n = 19

Recommendations on therapy were given in 18/21 discussions (85.7%) and recommendations on diagnostic procedures in 14/21 discussions (66.7%; Supplementary Table S4). In two cases, tumor material or CSF supernatant were shipped to another EU country for second opinion respectively further neuropathological diagnostics following recommendation in the VMTB. Recommendations were implemented, at least partly, in 94.7% of cases (n = 18/19, Fig. 2a). In 15/19 cases, all recommended measures were adopted. In three cases, not all recommendations were followed: In one case, a recommended review of neuropathology was not possible due to lack of tumor material. In two cases, recommendations were broad and encompassed different possible treatment strategies and therefore could not all be implemented. In one case, recommendations were not implemented due to severe unexpected clinical deterioration after the tumor board discussion.

### Knowledge of and satisfaction with the ERN PaedCan CNS tumor board

The majority of inquiring physicians learnt about the ERN PaedCan CNS tumor board from colleagues (77.8%, n = 7/9). Conferences, and the ERN and CPMS websites led to public notice of the tumor board less frequently. None of the inquiring physicians learnt about the tumor board through a virtual newsletter (Supplementary Fig. 1).

The satisfaction of the participants with the ERN PaedCan CNS tumor board was high. The recommendations were considered helpful in 89.5% (n = 17/19, Fig. 2b). All participants would present other patients in the ERN PaedCan CNS tumor board and recommend the tumor board to colleagues. More than half of the inquiring physicians (55.5%, n = 5/9) used an open text box to express their satisfaction with the tumor board organization and documentation.

The main obstacle were technical issues regarding data provision, especially upload of DICOM images, which was mentioned by 55.6% of inquiring physicians (Supplementary Table S5). Other obstacles concerned the complexity of CPMS management, the tight time frame for data provision, and the administrative effort of tumor board preparation. In two cases, the VMTB provided multiple treatment suggestions overwhelming the inquiring physician’s decision making. When asked for suggestions on how the tumor board conduct and recommendations could be improved, technical improvement of both the virtual video conference (sound quality) as well as the CPMS, especially upload of MRI DICOM data, were mentioned.

## Discussion

Previous reports show that consultations in multidisciplinary tumor boards can sustainably improve the care of children with CNS tumors in low- and middle-income countries [6, 7, 16], and may also lead to enhanced outcome in patients with rare pediatric tumors in high-income countries [17]. We report our experience of implementing a regular European VMTB for pediatric CNS tumors within ERN PaedCan. The results of our study demonstrate that an ERN PaedCan CNS tumor board is feasible and perceived as useful by the great majority of participants.

All EU countries are considered high-income on a global level [14], yet the economic and structural resources available for diagnostics and treatment in pediatric oncology are unequally distributed within the EU. This is especially relevant for molecular diagnostics: The fifth WHO classification of CNS tumors from 2021 strengthens the role of molecular diagnostics and underlines the significance of integrated diagnoses [18], marking a new era of neuro-oncology in which advanced neuropathological techniques such as DNA methylation profiling [19] and targeted next-generation sequencing [20] have direct implications for diagnosis and grading, and thus patient care. The importance of integrated histological and molecular diagnoses as crucial diagnostic assessments in pediatric neuro-oncology has been recently underlined by the SIOPE-BTG [21]. However, not all EU countries have the clinical and scientific structures in place to provide pediatric neuro-oncological patients with all of these molecular diagnostic tools, and also interpretation of their results requires specialized expertise. Our experience shows that the ERN PaedCan CNS tumor board can help to connect countries with fewer structural resources with experts from other EU countries, hereby helping to identify patients with the highest need to ship tumor material for neuropathological review including molecular profiling and arranging individual solutions for selected patients. Independent from molecular diagnostics, discussions were perceived as highly beneficial also by physicians from larger, resourceful centers, who participated in the VMTB. This demonstrates that CBHC for children with CNS tumors can be valuable independent of actual center size. Importantly, the VMTB connects professionals, who do not necessarily have been in contact previously. By bringing together professionals from different EU countries dedicated to pediatric neuro-oncology, all with different levels of experience and focus of expertise, the VMTB also establishes a platform for cross-border collaboration and possibly even research beyond the single case.

Multicenter trials, in which retrospective central neuroradiographic review was conducted, showed that incorrect or incomplete staging was associated with a lower than expected survival [22–24]. Real-time implementation of central review of assessments fundamental for staging is therefore an important tool to increase quality and accuracy of staging in the context of clinical trials. It is reasonable to assume that patients outside of clinical trials also benefit from systematic, centralized review. In most countries presenting patients in the ERN PaedCan CNS tumor board, there is no established structured review system available. The review of imaging by a neuroradiologist from the neuroradiological reference center for the HIT studies of the GPOH is therefore one major advantage of the ERN PaedCan CNS tumor board.

Technical issues were a particular challenge during the pilot phase of the VMTB. Upload of MRI DICOM data was time-consuming and in two cases even impossible. The resolution of those issues is crucial for continuance of the format, as inquiring and consulting physicians make time for the VMTB discussions in addition to their demanding daily clinical duties. This study did not assess the preparation time, but former studies indicate that preparation time for MTB show high variability [25] and can be a significant time burden, especially for the neuroradiologist [26]. When technical problems in downloading DICOM data add on top of the actual time spent evaluating before and presenting MRI images during the VMTB, the conduct of the VMTB might become impossible for the neuroradiologist due to existing time limitations. Providentially, the EU is currently working on an improved CPMS 2.0, which is expected to provide a more user-friendly experience. Adequate resources are fundamental for the conduct and maintenance of CBHC, as demonstrated by the high activity of the Lithuanian center, which was supported by the EU funded twinning project TREL (Twinning in Research and Education to improve survival in childhood solid tumors in Lithuania) [27]. Personal effort and time are required for both organization of the VMTB and from the experts discussing complex cases. Fortunately, the VMTB has received follow-up funding and is expected to be continued with the release of the updated CPMS 2.0. In the pilot phase, the tumor board was not directed at broader education of European healthcare professionals. For the resumption of the VTMB, it might be beneficial to provide access to the VTMB for children with CNS tumors to a broader audience of healthcare professionals for educational purposes.

Our study has the following limitations: firstly, physicians were asked to complete the survey after the end of the pilot phase. Due to the time interval between discussion and survey, the responses might have been affected by recall bias. Secondly, our survey did not assess parameters such as preparation time or change of treatment, and did not address the consulting experts but only the inquiring physician. Further research is needed to assess the prospects and challenges of a European tumor board for children with CNS tumors. Finally, the pilot phase covered only a limited period and cases. Though patient numbers of the pilot phase are comparable to those reported from other VMTB formats for pediatric oncology [4, 28], measures to increase participation in the future are desirable. These include but are not limited to improved dissemination of knowledge of the board and implementation of an improved, swift technical process with the CPMS 2.0. We expect participation to increase over time, as observed in other virtual, pan-institutional tumor boards for children with cancer [4, 5, 28]. Despite these limitations, the findings of the pilot phase encourage us to continue and further develop the ERN PaedCan CNS tumor board.

In conclusion, the pilot phase of the new European ERN PaedCan CNS tumor board proves that a pan-European tumor board for pediatric neuro-oncology can be implemented and potentially improves the clinical care for children with CNS tumors by offering real CHBC and professional communication on a European level independent of personal contacts.

## Supplementary Information

Below is the link to the electronic supplementary material.

Supplementary file1 (PPTX 46 kb)

Supplementary file2 (DOCX 29 kb)

## Data Availability

Raw data is available upon reasonable request from the corresponding author, provided ethical and legal requirements are met.
