# Supplementary material for: One-year follow-up of the new European reference network for pediatric cancers (ERN PaedCan) tumor board for pediatric CNS tumors: lessons learnt and future prospects
Source: J Neurooncol. 2025 Sep 16;175(3):1415–23. doi: 10.1007/s11060-025-05189-5 (PMC12511263; doi:10.1007/s11060-025-05189-5)
Supplement: Supplementary file 1 — Supplementary file1 (PPTX 46 kb) [file 11060_2025_5189_MOESM1_ESM.pptx]

## Slide 1
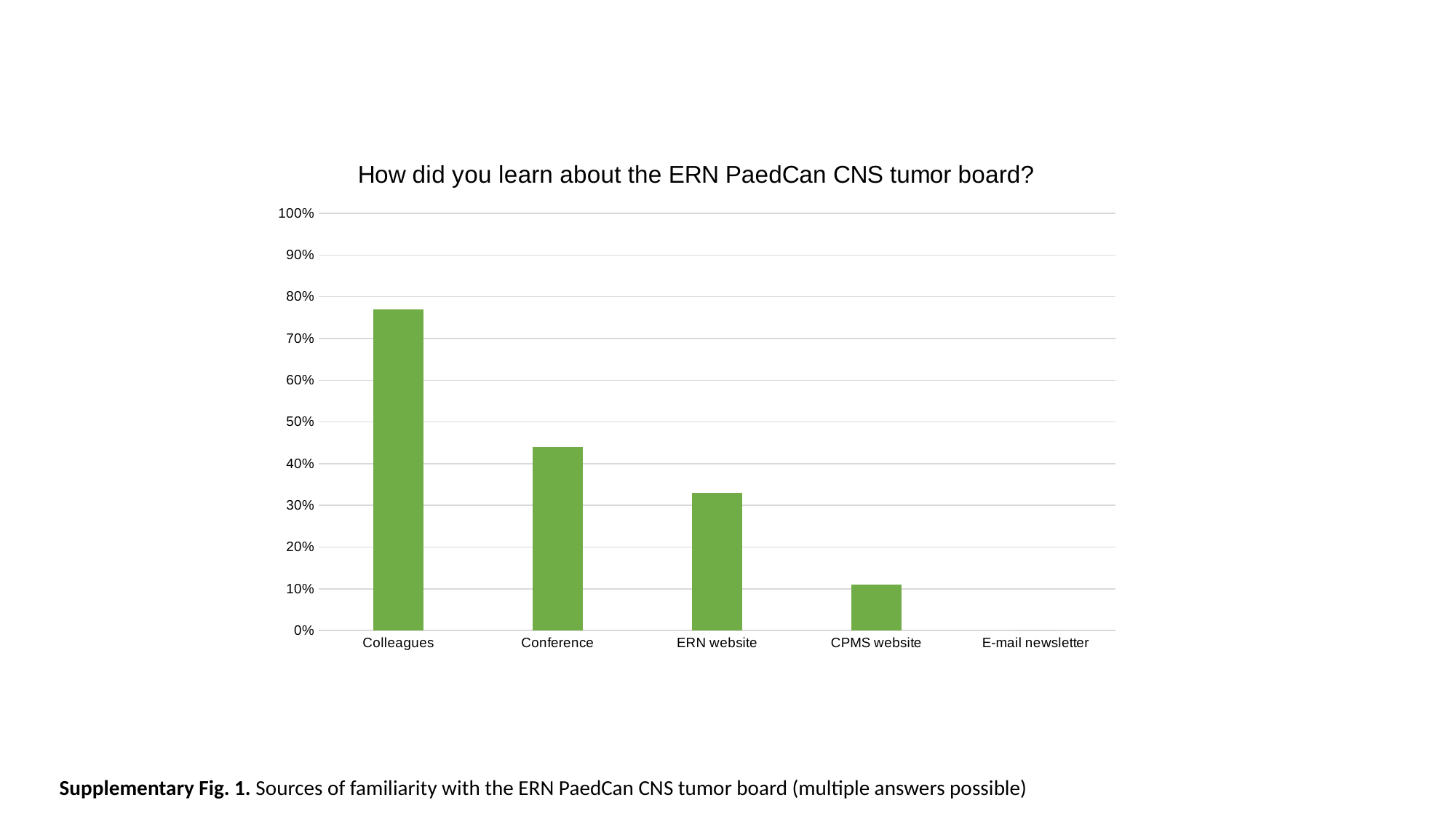

### Chart: How did you learn about the ERN PaedCan CNS tumor board?
| Category | |
|---|---|
| Colleagues | 0.77 |
| Conference | 0.44 |
| ERN website | 0.33 |
| CPMS website | 0.11 |
| E-mail newsletter | 0.0 |Supplementary Fig. 1. Sources of familiarity with the ERN PaedCan CNS tumor board (multiple answers possible)
