# Supplementary material for: One-year follow-up of the new European reference network for pediatric cancers (ERN PaedCan) tumor board for pediatric CNS tumors: lessons learnt and future prospects
Source: J Neurooncol. 2025 Sep 16;175(3):1415–23. doi: 10.1007/s11060-025-05189-5 (PMC12511263; doi:10.1007/s11060-025-05189-5)
Supplement: Supplementary file 2 — Supplementary file2 (DOCX 29 kb) [file 11060_2025_5189_MOESM2_ESM.docx]

**Supplementary Material**

**Supplementary Table S1. Conduct of and specialties involved in institutional tumor boards and availability of national quality control (n=9)**

| **Institutional tumor boards are …** | **n** | **%** |
| --- | --- | --- |
| Conducted regularly with fixed time slots | 3 | 33.3 |
| Conducted on a case-by-case basis | 6 | 66.7 |
| Not available | 0 | 0 |
| **Specialties regularly involved in institutional tumor boards** |  |  |
| Pediatric oncology | 9 | 100 |
| Radiotherapy | 6 | 66.6 |
| Neuroradiology | 6 | 66.6 |
| Radiology (without sub-specialization for neuroradiology) | 5 | 55.6 |
| Neurosurgery | 9 | 100 |
| Neuropathology | 5 | 55.6 |
| Pathology (without sub-specialization for neuropathology) | 3 | 33.3 |
| **National central review for pediatric patients with CNS tumors is …** |  |  |
| Available for neuropathology, CSF cytology and imaging | 2 | 22.2 |
| Available for neuropathology | 1 | 11.1 |
| Not available | 6 | 66.7 |

**Supplementary Table S2. Attendance of and specialties involved in the virtual ERN PaedCan CNS tumor board discussion**

| **Tumor board ID** | **Number of attending experts** | **Specialties of attending experts** | **Number of attendees from inquiring institution** | **Specialties of attendees of the inquiring institution** | **Total number of participants** | **Number of specialties present** |
| --- | --- | --- | --- | --- | --- | --- |
| 1 | 5 | Pediatric oncology, Neuroradiology | 3 | Pediatric oncology | 8 | 2 |
| 2 | 7 | Pediatric oncology, Neuroradiology, Radiotherapy, Neurosurgery | 15 | Pediatric oncology, Radiology, Radiotherapy, Neurosurgery, Ophthalmology | 22 | 5 |
| 3 | 8 | Pediatric oncology, Neuroradiology, Radiotherapy, Neurosurgery | 15 | Pediatric oncology, Radiology, Radiotherapy, Neurosurgery, Ophthalmology | 23 | 5 |
| 4 | 7 | Pediatric oncology, Neuroradiology, Radiotherapy, Neurosurgery | 15 | Pediatric oncology, Radiology, Radiotherapy, Neurosurgery, Ophthalmology | 22 | 5 |
| 5 | 7 | Pediatric oncology, Neuroradiology, Neuropathology | 8 | Pediatric oncology, Radiology, Neuropathology | 15 | 3 |
| 6 | 6 | Pediatric oncology, Neuroradiology | 8 | Pediatric oncology, Radiology, Radiotherapy, Neurosurgery, Neuropathology | 14 | 5 |
| 7 | 8 | Pediatric oncology, Neuroradiology, Radiotherapy, Neurosurgery | 6 | Pediatric oncology, Neuroradiology, Radiotherapy, Neuropathology | 14 | 5 |
| 8 | 8 | Pediatric oncology, Neuroradiology, Radiotherapy, Neurosurgery | 9 | Pediatric oncology, Neuroradiology, Radiotherapy, Neuropathology | 17 | 5 |
| 9 | 7 | Pediatric oncology, Neuroradiology | 5 | Pediatric oncology, Pathology | 12 | 3 |
| 10 | 6 | Pediatric oncology, Neuroradiology, Radiotherapy | 10 | Pediatric oncology, Radiotherapy, Neuropathology | 16 | 4 |
| 11 | 8 | Pediatric oncology, Neuroradiology, Radiotherapy | 3 | Pediatric oncology | 11 | 3 |
| 12 | 6 | Pediatric oncology, Neuroradiology | 1 | Pediatric oncology | 7 | 2 |
| 13 | 8 | Pediatric oncology, Neuroradiology | 2 | Pediatric oncology | 10 | 2 |
| 14 | 8 | Pediatric oncology, Neuroradiology, Radiotherapy | 2 | Pediatric oncology | 10 | 3 |
| 15 | 10 | Pediatric oncology, Neuroradiology, Radiotherapy | 4 | Pediatric oncology | 14 | 3 |
| 16 | 6 | Pediatric oncology, Neuroradiology | 1 | Pediatric oncology | 7 | 2 |
| 17 | 4 | Pediatric oncology, Neuroradiology | 11 | Pediatric oncology, Neuroradiology, Neurosurgery, Molecular biology | 15 | 4 |
| 18 | 4 | Pediatric oncology, Neuroradiology | 1 | Pediatric oncology | 5 | 2 |
| 19 | 9 | Pediatric oncology, Neuroradiology, Neuropathology | 1 | Pediatric oncology | 10 | 3 |
| 20 | 6 | Pediatric oncology, Neuroradiology | 6 | Pediatric oncology, Radiotherapy, Neuropathology | 12 | 4 |
| 21 | 5 | Pediatric oncology, Neuroradiology | 4 | Pediatric oncology, Neuroradiology, Radiotherapy | 9 | 3 |

**Supplementary Table S3. Discordances between neuroradiological assessment by German national reference center and local institution (n = 19)**

| **Discordance in neuroradiological assessment** | | | **n** | **%** |
| --- | --- | --- | --- | --- |
| Yes | | | 4 | 21.1 |
| No | | | 15 | 78.9 |
| **Description of discordance between assessments** | | | | |
| **Timepoint** | **Local institution** | **German reference center for neuroradiology** | | |
| Initial diagnosis | Presence of two supratentorial lesions | Presence of two supratentorial lesions and one infratentorial lesion | | |
| After surgery | No residual tumor | Tumor residue of metastases | | |
| At relapse | Metastases along the surgical access | Metastases along the surgical access and new lesions supratentorial without proximity to surgical access | | |
| After surgery | No residual tumor | Small residual tumor at borders of resection cavity | | |

**Supplementary Table S4. Recommendations given according to category**

| **Recommendations** | **Number of tumor boards in which recommendation was given**  **(total = 21)** | **%** |
| --- | --- | --- |
| **Diagnostic** | 14 | 66.7 |
| - Completion of (re-)staging due to incomplete tests (i.e. no CSF cytology, no spinal MRI conducted) | 2 | 9.5 |
| - Repetition of imaging | 2 | 9.5 |
| - Conduct and timing of re-staging during therapy | 2 | 9.5 |
| - Assessments during follow-up | 2 | 9.5 |
| - Genetic testing | 3 | 14.3 |
| - Genetic testing not indicated | 1 | 4.8 |
| - Biopsy and pathological analysis | 1 | 4.8 |
| - Molecular analysis of tumor tissue | 2 | 9.5 |
| - Pathology review | 3 | 14.3 |
| - Elaborate testing (i.e. in vitro radiosensitivity) due to unexpected treatment-associated toxicity | 1 | 4.8 |
| - Additional diagnostic tests not indicated | 1 | 4.8 |
| **Therapeutic** | 18 | 85.7 |
| - Chemotherapy | 10 | 76.8 |
| - Radiotherapy | 6 | 28.6 |
| - Watch and wait | 3 | 14.3 |
| - Consideration of inclusion in clinical | 2 | 9.5 |
| - Re-surgery | 1 | 4.8 |
| - Palliative situation: Highly experimental therapy theoretically possible with limited evidence; discontinuation of therapy and best supportive care also an option | 2 | 9.5 |
| - Discontinuation of targeted therapy due to side effects | 1 | 4.8 |
| - Re-discussion in ERN PaedCan CNS tumor board after application of therapy | 2 | 9.5 |

**Supplementary Table S5. Obstacles observed during the process (n = 9)**

| Obstacle | n | % |
| --- | --- | --- |
| Providing MRI images via the CPMS | 5 | 55.6 |
| Immense administrative effort for preparation of the tumor board | 1 | 11.1 |
| Tight time frame for information upload | 1 | 11.1 |
| Time from initial request to video conference rather long | 1 | 11.1 |
| Navigation on the CPMS website | 2 | 22.2 |
| Limited time slots for video conference | 1 | 11.1 |
